# Severe Maternal Morbidity Among Pregnant People With Opioid Use Disorder Enrolled in Medicaid

**DOI:** 10.1001/jamanetworkopen.2024.53303

**Published:** 2025-01-07

**Authors:** Samantha G. Auty, Austin B. Frakt, Paul R. Shafer, Michael D. Stein, Sarah H. Gordon

**Affiliations:** 1 Department of Health Law, Policy and Management, Boston University School of Public Health, Boston, Massachusetts; 2 Partnered Evidence-Based Policy Resource Center, Veterans Administration Boston Healthcare System, Jamaica Plain, Massachusetts; 3 Department of Health Policy and Management, Harvard T.H. Chan School of Public Health, Boston, Massachusetts

## Abstract

**Importance:**

Pregnant people with opioid use disorder (OUD) are at high risk for potentially avoidable maternal morbidity. The majority of pregnant people with OUD receive health insurance through state Medicaid programs, but there is little comprehensive data on the burden of severe maternal morbidity (SMM)—a composite measure of adverse maternal health outcomes—among this high-risk group.

**Objective:**

To estimate rates of SMM among Medicaid-enrolled pregnant people with OUD from 2016 to 2018.

**Design, Setting, and Participants:**

Using the Transformed Medicaid Statistical Information System Analytic Files, this cross-sectional study identified 96 309 pregnant people with OUD enrolled in Medicaid in 47 states with 108 975 deliveries between March 1, 2016, and November 16, 2018. Data were analyzed from August 1, 2023, to September 1, 2024.

**Main Outcome and Measures:**

SMM was identified using *International Statistical Classification of Diseases and Related Health Problems, Tenth Revision* diagnosis and procedure codes for 20 relevant conditions and was measured per 10 000 live births nationally and by state. Rates of SMM were also stratified by the timing of Medicaid enrollment before delivery.

**Results:**

From 2016 to 2018, 96 309 Medicaid enrollees had a diagnosis of OUD before a live birth (108 975 deliveries). The mean (SD) age of Medicaid-enrolled pregnant people with OUD was 28.8 (5.0) years. The mean (SD) rate of OUD among pregnant people enrolled in Medicaid was 324.8 (260.9) per 10 000 live births across states. Among this group, the mean (SD) unadjusted rate of SMM excluding blood transfusions among those with OUD was 292.1 (112.3) per 10 000 live births, with these rates varying substantially across states, from 101.0 per 10 000 live births in South Dakota to 682.2 per 10 000 live births in California. Adjustment for enrollee characteristics and comorbidities did not meaningfully alter the estimated rate of SMM (305.6 [95% CI, 245.2-408.2] per 10 000 live births). Rates of SMM generally increased with decreased durations of Medicaid enrollment.

**Conclusions and Relevance:**

This cross-sectional study of pregnant people enrolled in Medicaid found that the rate of OUD among this group was more than twice as high as previous estimates. Pregnant people with OUD face a disproportionately high risk of SMM, particularly those who enroll in Medicaid later in pregnancy. Targeted interventions that facilitate early Medicaid enrollment and coverage continuity may be needed to reduce the burden of adverse outcomes in this group.

## Key Points

**Question:**

Nationally and at the state level, what are the rates of opioid use disorder (OUD) among Medicaid-enrolled pregnant people, and what are the rates of severe maternal morbidity (SMM) among this group?

**Findings:**

Using the Transformed Medicaid Statistical Information System Analytic Files, this cross-sectional study identified 96 309 pregnant people with OUD enrolled in Medicaid in 47 states with 108 975 deliveries between March 1, 2016, and November 16, 2018.

**Meaning:**

These findings suggest that rates of OUD among Medicaid-enrolled pregnant people are higher than previously estimated, as are rates of SMM, and evidence-based solutions are needed to improve the health of pregnant people with OUD.

## Teaser

This cross-sectional study estimates rates of severe maternal morbidity among Medicaid-enrolled pregnant people with opioid use disorder from 2016 to 2018.

## Introduction

Rates of opioid use disorder (OUD) among pregnant people have increased considerably over the past several decades,^1^ and this group faces a disproportionately high risk of adverse maternal health outcomes.^2^ OUD is a risk factor for severe maternal morbidity (SMM),^2^ a composite measure of adverse pregnancy and delivery complications that can result in serious health consequences or mortality.

Insuring more than 70% of pregnant people with OUD,^2^ Medicaid is the primary source of coverage through which pregnant and postpartum people with OUD access evidence-based services that can reduce the risk of poor maternal health outcomes.^3^ Many people gain Medicaid eligibility as a function of pregnancy, and state Medicaid programs are required to cover prenatal care and delivery services for those with incomes below 133% of the federal poverty level through at least 60 days post partum.^4^

Despite being the dominant coverage mechanism for pregnant people with OUD,^2^ very little is known about the prevalence of OUD among pregnant people enrolled in Medicaid or the burden of adverse maternal health outcomes in this population. Most previous work has relied on administrative claims data from inpatient hospitalizations,^2,5^ which may underestimate the true burden of OUD given that utilization of services related to substance use most often occurs in outpatient settings.^6^ A smaller body of research has assessed SMM among those with OUD using state-specific all-payer claims data, but results may not generalize to other states.^2,5,7^ In this study, we use the Transformed Medicaid Statistical Information System Analytic Files (TAF), which contain inpatient and outpatient claims data for all Medicaid enrollees in all 50 states and the District of Columbia, to assess rates of OUD among those with Medicaid-paid births and to measure their rates of SMM.

## Methods

### Data

The data source was an extract from the TAF from the Centers for Medicare & Medicaid Services from 2016 to 2018. The extract included claims for enrollees with any *International Statistical Classification of Diseases and Related Health Problems, Tenth Revision* (*ICD-10*) diagnosis or procedure codes or diagnostic related group codes indicative of the prenatal or postpartum period, delivery, maternal health conditions, or obstetric-related services from all 50 states and the District of Columbia. For this study, we had access to the Inpatient (IP) files, the Other Services (OT) files, and the Demographic and Eligibility (DE) files.

The IP and OT files contain claims-based health service encounter information, including *ICD-10* diagnosis and procedure codes, in addition to the dates of service and the service setting (eg, hospital or office). The IP files contain claims from inpatient hospital services, whereas the OT files contain a mix of claims from outpatient (eg, office-based services and outpatient specialty services) and inpatient hospital services. The extent to which hospitalization claims are contained in the OT file varies across states. The DE file contains data on enrollee eligibility characteristics (eg, enrollment dates and eligibility mechanism), enrollee demographic characteristics (eg, age, gender, residence, and race and ethnicity), and Medicaid plan characteristics (eg, plan type). The completeness of enrollee characteristics, such as race and ethnicity, income, and marital status, vary across states and over time owing to differences in enrollment practices across state Medicaid programs. All files can be linked using the unique beneficiary identification number, which is consistent across states and years. We excluded New Hampshire, Rhode Island, Pennsylvania, and the District of Columbia owing to data-quality issues in these states. This cross-sectional study was approved by the Boston University institutional review board and followed the Strengthening the Reporting of Observational Studies in Epidemiology (STROBE) reporting guideline.

### Sample

The sample included all enrollees with Medicaid-paid live births between March 1, 2016, and November 16, 2018, to observe sufficient data before and after delivery. Live births were identified using a previously validated method.^8^ Multiple deliveries from a single enrollee were included when they were separated by at least 180 days. During the study period, 4 412 067 live births were identified in the TAF data.

OUD was identified using a previously validated method using *ICD-10* diagnosis codes (eMethods and eTable in Supplement 1).^9^ We considered enrollees as having OUD if they had any qualifying diagnosis code before delivery. Notably, we did not use codes for neonatal abstinence syndrome to identify OUD. Use of these codes may inappropriately identify those with other substance use disorders (SUDs) or those who take medications that cause withdrawal symptoms in infants as having OUD. Rates were calculated as the number of enrollees with OUD per 10 000 live births.

### Primary Outcome

SMM within 6 weeks of delivery was identified using *ICD-10* diagnosis and procedure codes for 20 conditions (eg, sepsis, shock, and acute myocardial infarction) compiled by the Centers for Disease Control and Prevention that represent adverse outcomes that may result in long- or short-term health consequences or maternal mortality without adequate treatment.^10^ Observations in which a blood transfusion was the only indicator of SMM were excluded, given evidence that blood transfusion alone may not represent a truly severe event.^11^ The SMM measure was designed for use with inpatient claims data,^12^ and its application to outpatient claims has not been validated. To comport with previously validated methods while simultaneously leveraging the breadth of claims in the TAF data, SMM was measured using hospital and emergency department claims in the OT files and all claims in the IP file, which contain only claims from inpatient hospital service use. Rates of SMM were calculated as the number of enrollees who experienced SMM per 10 000 live births.

### Covariates

Delivery-level covariates included age, mode of delivery, gestational age at delivery, and health status. Age was calculated as the age at the date of delivery. Race and ethnicity data were obtained from the TAF DE files; the completeness of race and ethnicity data varies across states because of differences in Medicaid enrollment practices—not all states require enrollees self-report race and ethnicity at the time of Medicaid enrollment. As such, race and ethnicity were measured as the first nonmissing observation per enrollee. Health status was measured using the Quan-Elixhauser Comorbidity Index summary score,^13^ which ranges from 0 to 31, with higher scores indicating worse health status. The Quan-Elixhauser Comorbidity Index summary score was generated using claims data from the IP and OT files in the 90 days before delivery. Mode of delivery (cesarean, vaginal, or unknown)^14^ and gestational age at delivery^15^ were identified using established methods that use *ICD-10* procedure and diagnosis codes on the delivery claim. Indicators of other SUDs were also generated using claims data from the IP and OT files in the 90 days before delivery.

### Statistical Analysis

The characteristics of pregnant people with OUD enrolled in Medicaid were examined using *t* tests and χ^2^ tests. Next, we generated unadjusted rates of SMM per 10 000 live births by state. We then generated adjusted rates of SMM using logistic regression. Rates were adjusted for age, race and ethnicity, Quan-Elixhauser composite score, mode of delivery, gestational age at delivery, the presence of other SUDs, and Medicaid plan type (ie, fee-for-service vs comprehensive Medicaid managed care). Year fixed effects were used to control for secular trends. Using this model, we generated adjusted predicted probabilities of SMM at the enrollee-delivery level. Estimated probabilities were then summed to the state of delivery to generate state-level rates.

We also examined whether rates of SMM differed by the timing of Medicaid enrollment. For this analysis, we limited the sample to enrollees who delivered after January 1, 2017, to allow for a full year of observation before delivery. We then identified the earliest occurrence of enrollment, allowing for gaps in coverage of 31 days or less. Enrollment was categorized as occurring before pregnancy, in the first trimester, in the second trimester, in the third trimester, or at delivery (defined as enrollment occurring within 7 days of delivery). For all analyses, results were considered statistically significant if the associated *P* value was less than .05. All analyses were conducted at the delivery level in Stata MP, version 17.0 (StataCorp LLC).

## Results

### Sample Characteristics

**Figure 1.**
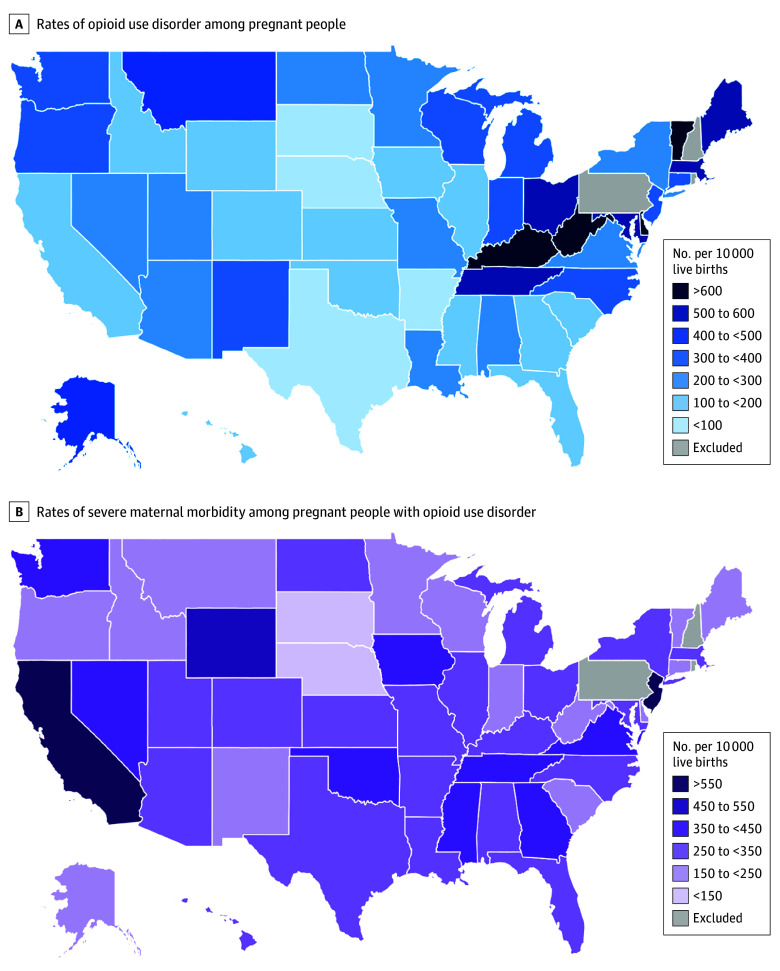
Rates of Opioid Use Disorder and Severe Maternal Morbidity Among Pregnant People With Opioid Use Disorder Per 10 000 Live Medicaid-Paid Births

From March 1, 2016, to November 16, 2018, 96 309 Medicaid enrollees had a diagnosis of OUD before a live birth (108 975 deliveries). Across states, the mean (SD) rate of OUD among pregnant people enrolled in Medicaid was 324.8 (260.9) per 10 000 live births, but rates varied substantially across states (Figure 1A). Of 108 975 deliveries to pregnant people with OUD, 42 265 (38.8%) had another SUD. The most common comorbid substances used were cannabis (18.0% [n = 19 640]), sedatives (15.1% [n = 16 423]), stimulants (10.9% [n = 11 977]), cocaine (10.4% [n = 11 300]), and alcohol (9.6% [n = 8097]). The mean (SD) age of Medicaid-enrolled pregnant people with OUD was 28.8 (5.0) years. Regarding race and ethnicity, deliveries in the sample were to enrollees who were 7.3% (n = 7082) Hispanic, 7.9% (n = 7627) non-Hispanic Black, 69.6% (n = 67 015) non-Hispanic White, and 4.0% (n = 3874) non-Hispanic other race and ethnicity (ie, Asian, American Indian or Alaska Native, Hawaiian or Other Pacific Islander, and multiracial), and 11.1% (n = 10 711) enrollees had missing race and ethnicity data. At delivery, most enrollees received Medicaid benefits through a comprehensive Medicaid managed care plan (73.9% [n = 80 514]). See the Table for full sample characteristics.

**Table.** Demographic and Health Characteristics at Delivery Among Pregnant People With Opioid Use Disorder Enrolled in Medicaid From 2016 to 2018

Characteristic	Enrollee, No. (%)^a^
Demographic	
Age, mean (SD), y	28.8 (5.0)
Race and ethnicity	
Non-Hispanic Black	7627 (7.9)
Hispanic	7082 (7.3)
Non-Hispanic White	67 015 (7.3)
Non-Hispanic other^b^	3874 (4.0)
Missing	10 711 (11.1)
Timing of Medicaid enrollment^c^	
Before pregnancy	58 687 (74.2)
First trimester	11 104 (14.0)
Second trimester	5325 (6.7)
Third trimester	3173 (4.0)
At delivery	755 (1.0)
Medicaid plan type	
Medicaid managed care	80 514 (73.9)
Medicaid fee for service	28 461 (26.1)
Health and delivery	
Mode of delivery	
Vaginal	72 869 (66.7)
Cesarean	33 425 (30.7)
Unknown	2861 (2.6)
Quan-Elixhauser Comorbidity Index score, mean (SD)	2.1 (1.9)
Cannabis use disorder	19 640 (18.0)
Sedative use disorder	16 423 (15.1)
Stimulant use disorder	11 977 (10.9)
Cocaine use disorder	11 300 (10.4)
Alcohol use disorder	8097 (9.6)

^a^ Percentages may not sum to 100% because of rounding.

^b^ The groups included were Asian, American Indian or Alaska Native, Hawaiian or Other Pacific Islander, and multiracial. Race and ethnicity of enrollees were captured as the first nonmissing value across all deliveries.

^c^ Timing of Medicaid enrollment was examined among enrollees who had a live birth after January 1, 2017 (enrollees = 71 353, deliveries = 79 044), to allow for 1 year of observation to adequately assess enrollment timing.

### Severe Maternal Morbidity

Across states, the mean (SD) rate of SMM excluding blood transfusions among Medicaid enrollees with OUD was 292.1 (112.3) per 10 000 live births. Among those who experienced SMM (n = 3644), the 5 most common SMM conditions were adult respiratory distress syndrome (23.2% [n = 845]), sepsis (14.1% [n = 514]), ventilation (13.8% [n = 502]), pulmonary edema or acute heart failure (13.4% [n = 489]), and eclampsia (12.9% [n = 471]). Rates of SMM per 10 000 live births varied substantially across states (Figure 1B), ranging from 101.0 in South Dakota to 682.2 in California. Adjustment did not meaningfully alter rates of SMM across states, producing an adjusted rate of SMM of 305.6 (95% CI, 245.2-408.2) per 10 000 live births.

**Figure 2.**
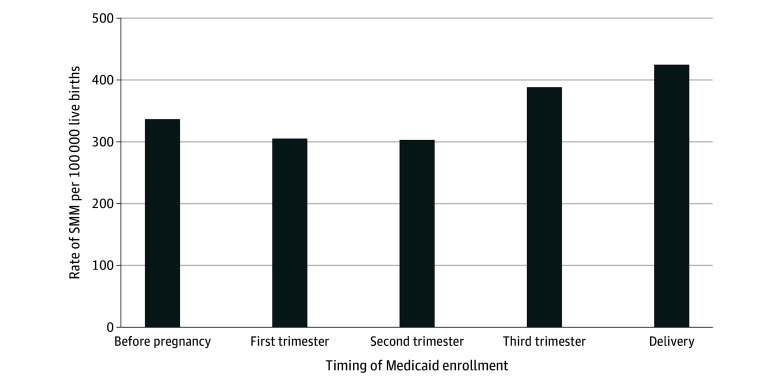
Rate of Severe Maternal Morbidity (SMM) Among Pregnant People With Opioid Use Disorder Per 10 000 Live Medicaid-Paid Births Stratified by Timing of Medicaid Enrollment This analysis was conducted in a sample of enrollees who delivered after January 1, 2017 (enrollees = 71 353, deliveries = 79 044), to observe prepregnancy Medicaid enrollment and to adequately assess enrollment timing.

Among enrollees who delivered after January 1, 2017, the mean (SD) duration of Medicaid enrollment before delivery was 17.4 (9.1) months. Rates of SMM generally increased with decreased durations of Medicaid enrollment (Figure 2). Notably, the rate of SMM was 335.7 per 10 000 live births (n = 1970) among those who enrolled in Medicaid before pregnancy, whereas the rate of SMM among those who enrolled at delivery was 423.8 per 10 000 live births (n = 32).

## Discussion

In this study using nationally representative Medicaid data from 2016 to 2018, we found that the burden of OUD among pregnant people enrolled in Medicaid was substantially higher than previously estimated.^1,16^ Extant estimates of OUD among Medicaid-enrolled pregnant people were 146 per 10 000 live births, 75% below our estimate of 324 per 10 000 live births.^16^ Our estimates are likely higher than prior work because TAF data allow for observation of both inpatient and outpatient care, enabling the capture of outpatient SUD treatment services. The majority of SUD treatment occurs in outpatient settings,^17^ thus, use of inpatient data alone may underestimate the burden of OUD.

We observed large differences in rates of SMM to deliveries among pregnant people with OUD across states. Consistent with our findings, other studies using TAF have documented similar state-by-state differences in SMM rates among Medicaid-enrolled pregnant people.^18^ Differences in SMM may be partially attributable to state-level differences in Medicaid policies that affect enrollment and maintenance of coverage, Medicaid benefit design, and health care capacity in addition to enrollee-level factors that may influence risk of SMM.^18^ Prenatal and postpartum care and medications for OUD (MOUD) mitigate the risk of adverse maternal health outcomes,^19,20^ yet less than half of pregnant people with OUD use these services before delivery.^19^ Moreover, coverage of MOUD varies across states. Although most state Medicaid fee-for-service and Medicaid managed care plans cover all types of MOUD, plans use varying utilization management strategies to control access to these services. Therefore, the relative ease that enrollees can access MOUD may also be associated with SMM. Relatedly, Medicaid coverage for substance use treatment services may affect the detection of OUD diagnoses in administrative claims data. The generosity of this coverage might affect how often enrollees seek OUD-related services, thereby influencing researchers’ ability to identify enrollees with OUD in claims data.

Other state policies, such as those that criminalize substance use during pregnancy or those that classify substance use during pregnancy as child abuse, might also affect detection of OUD and, subsequently, SMM in this group of pregnant people.^21^ In states with these policies, fear of legal repercussions related to opioid use is a substantial barrier to seeking guideline-concordant care for OUD, such as MOUD.^22^ Notably, in several states with such laws in place during our study period^23^—such as Texas—we found lower than average rates of OUD but higher than average rates of SMM. Other work has found that punitive policies related to substance use during pregnancy were associated with higher rates of neonatal abstinence syndrome, suggesting these policies might deter those with OUD from seeking evidence-based care during pregnancy that might reduce their long-term risk of adverse outcomes.^21,23^ Although the specific factors that drive state-level differences in rates of SMM among pregnant people with OUD are not examined in this study, the data might inform tailored interventions or policies to improve the health of this group of pregnant people across states.

### Limitations

This study has several limitations. Sample and outcome identification required that certain billing codes were present on Medicaid-paid claims; thus, we may be underestimating the rates of OUD and SMM among those whose care for these conditions was not financed by Medicaid. Our analysis is also limited to those who had a live birth, and SMM rates may differ among those whose pregnancy did not result in a live birth. To adequately assess the timing of Medicaid enrollment, we were only able to examine SMM rates stratified by enrollment timing in a subset of our sample. Owing to data quality, 3 states and the District of Columbia were excluded from analyses, and results may not generalize to those states.

## Conclusions

This cross-sectional study of pregnant people enrolled in Medicaid found that the rate of OUD among this group was over twice as high as previous estimates. The existing literature may have underestimated the prevalence of OUD among Medicaid-enrolled pregnant people, and this population faces a disproportionately high risk of SMM. State Medicaid programs are uniquely positioned to implement strategies that address the burden of SMM among pregnant people with OUD. Doing so will require early identification of pregnant Medicaid enrollees with OUD and implementation of targeted strategies to increase engagement with perinatal care and SUD treatment.

## Notes

Supplement 1.
eMethods.
**eTable.** ICD-10 Codes Used to Opioid Use Disorder

Supplement 2.
Data Sharing Statement
