# Supplementary material for: Severe Maternal Morbidity Among Pregnant People With Opioid Use Disorder Enrolled in Medicaid
Source: JAMA Netw Open. 2025 Jan 7;8(1):e2453303. doi: 10.1001/jamanetworkopen.2024.53303 (PMC11707626; doi:10.1001/jamanetworkopen.2024.53303)
Supplement: Supplement 1. — eMethods. eTable. ICD-10 Codes Used to Opioid Use Disorder [file jamanetwopen-e2453303-s001.pdf]

Supplemental Online Content

Auty SG, Frakt AB, Shafter PR, Stein MD, Gordon SH. Severe maternal morbidity among pregnant people with opioid use disorder enrolled in Medicaid. *JAMA Netw Open*. 2024;8(1):e2453303. doi:10.1001/jamanetworkopen.2024.53303

eMethods.

eTable. ICD-10 Codes Used to Opioid Use Disorder

This supplemental material has been provided by the authors to give readers additional information about their work.

© 2025 Auty SG et al. *JAMA Network Open.*

eMethods.

Measurement of Race and Ethnicity

Race and ethnicity data are collected as part of Medicaid enrollment in most states, though not all states require enrollees disclose this information at the time of eligibility determination. The TAF DE file contains expanded and condensed information on enrollee race and ethnicity. Owing to small group sizes, the condensed race and ethnicity variable was used in this study. This variable includes the following levels, which were pre-specified in the data: 1) white, non-Hispanic; 2) Black, non-Hispanic; 3) Asian, non-Hispanic; 4) American Indian and Alaskan Native, non-Hispanic; 5) Hawaiian or Pacific Islander, non-Hispanic; 6) multiracial, non-Hispanic, and 7) Hispanic. Based on the condensed race and ethnicity variable included in the TAF DE file, we generated a variable based on frequency with the following levels: non-Hispanic Black, non-Hispanic Other race, Hispanic, non-Hispanic white, or missing.

Outcome Measurement

Rates of OUD at the state and national level were estimated as follows:

$$\left(\frac{Number\ of\ Enrollees\ with\ OUD\ and\ a\ Medicaid\ Paid\ Live\ Birth}{Number\ of\ Enrollees\ with\ a\ Medicaid\ Paid\ Live\ Birth}\right) \times 10{,}000$$

Rates of SMM among those with OUD at the state and national level were estimated as follows:

$$\left(\frac{Number\ of\ Enrollees\ with\ OUD\ and\ Medicaid\ Paid\ Live\ Birth\ who\ Experience\ SMM}{Number\ of\ Enrollees\ with\ OUD\ and\ a\ Medicaid\ Paid\ Live\ Birth}\right) \times 10{,}000$$

© 2025 Auty SG et al. *JAMA Network Open.*

eTable. ICD-10 Codes Used to Opioid Use Disorder	
F11.10	Opioid abuse, uncomplicated
F11.120	Opioid abuse with intoxication, uncomplicated
F11.121	Opioid abuse with intoxication, delirium
F11.122	Opioid abuse with intoxication, with perceptual disturbance
F11.129	Opioid abuse with intoxication, unspecified
F11.14	Opioid abuse with opioid-induced mood disorder
F11.150	Opioid abuse with opioid-induced psychotic disorder, with delusions
F11.151	Opioid abuse with opioid-induced psychotic disorder, with hallucinations
F11.159	Opioid abuse with opioid-induced psychotic disorder, unspecified
F11.181	Opioid abuse with opioid-induced sexual dysfunction
F11.182	Opioid abuse with opioid-induced sleep disorder
F11.188	Opioid abuse with other opioid-induced disorder
F11.19	Opioid abuse with unspecified opioid-induced disorder
F11.20	Opioid dependence, uncomplicated
F11.21	Opioid dependence, in remission
F11.220	Opioid dependence with intoxication, uncomplicated
F11.221	Opioid dependence with intoxication, delirium
F11.222	Opioid dependence with intoxication, with perceptual disturbance
F11.229	Opioid dependence with intoxication, unspecified
F11.23	Opioid dependence with withdrawal
F11.24	Opioid dependence with opioid-induced mood disorder
F11.250	Opioid dependence with opioid-induced psychotic disorder, with delusions
F11.251	Opioid dependence with opioid-induced psychotic disorder, with hallucinations
F11.259	Opioid dependence with opioid-induced psychotic disorder, unspecified
F11.281	Opioid dependence with opioid-induced sexual dysfunction
F11.282	Opioid dependence with opioid-induced sleep disorder
F11.288	Opioid dependence with other opioid-induced disorder
F11.29	Opioid dependence with unspecified opioid-induced disorder
F11.920	Opioid use, unspecified with intoxication, uncomplicated
F11.921	Opioid use, unspecified with intoxication delirium
F11.922	Opioid use, unspecified with intoxication, with perceptual disturbance
F11.929	Opioid use, unspecified with intoxication, unspecified
F11.93	Opioid use, unspecified, with withdrawal
F11.94	Opioid use, unspecified, with opioid-induced mood disorder

© 2025 Auty SG et al. *JAMA Network Open.*

F11.950	Opioid use, unspecified with opioid-induced psychotic disorder, with delusions
F11.951	Opioid use, unspecified with opioid-induced psychotic disorder, with hallucinations
F11.959	Opioid use, unspecified with opioid-induced psychotic disorder, unspecified
F11.981	Opioid use, unspecified with opioid-induced sexual dysfunction
F11.982	Opioid use, unspecified with opioid-induced sleep disorder
F11.988	Opioid use, unspecified with other opioid-induced disorder
F11.99	Opioid use, unspecified, with unspecified opioid-induced disorder
Abbreviations: ICD-10-CM; international classification of disease tenth edition diagnosis code	

© 2025 Auty SG et al. *JAMA Network Open.*
