# Supplementary material for: Severe Maternal Morbidity Among Pregnant People With Opioid Use Disorder Enrolled in Medicaid
Source: JAMA Netw Open. 2025 Jan 7;8(1):e2453303. doi: 10.1001/jamanetworkopen.2024.53303 (PMC11707626; doi:10.1001/jamanetworkopen.2024.53303)
Supplement: Supplement 2. — Data Sharing Statement [file jamanetwopen-e2453303-s002.pdf]

Data Sharing Statement

Auty. Severe Maternal Morbidity Among Pregnant People With Opioid Use Disorder Enrolled in Medicaid. *JAMA Netw Open*. Published January 07, 2025.
doi:10.1001/jamanetworkopen.2024.53303

Data
Data available: No

Additional Information
Explanation for why data not available: Not publicaly available.
